# The Annual Meeting

**Published:** 1894-03

**Authors:** 


**The Annual Meeting** of the Texas State Medical Association will take place in Austin the last week in April (proximo), beginning Tuesday, April 24th, and holding four days. The

Committee of Arrangements, of which Dr. A. N. Denton is chairman, have been actively engaged, recently, in providing ways and means, and making up a program of the social, or outside, "functions." This program embraces an excursion to the famous dam and a steamboat trip on the magnificent new lake, a visit to the State University, where visitors will be entertained in an appropriate manner by the Faculty; a reception and collation at the Driskill, etc. That there will be "speech-making" may be taken for granted; but the probabilities are largely in favor of their being anything but "maudlin"; and it is safe to say there will be no "drinking bouts,"—though we believe that wine is in the program.

Dr. Swearingen, as chairman of the Committee on Transportation, has succeeded in securing the usual one and one-third fare rate, on the certificate plan, from all the roads terminating at Austin, and connecting lines agree to conform thereto; that is, the member or delegate purchases a ticket at starting point, paying full fare, and on the last day of the meeting, or, when he desires to return, he must have the Secretary of the Association to fill out and sign a certificate that will have been provided for the occasion, and which the ticket agent at the point of departure will furnish each purchaser of a ticket. On this certificate all the conditions on which a return fare of one-third may be procured are distinctly stated.

The following railroads have agreed to the one and one-third fare rate, on the certificate plan, and tickets will be on sale at all depots on all lines:

Gulf, Colorado & Sante Fe Railroad,
Sun Set—Southern Pacific Co.,
Missouri, Kansas & Texas Railroad,
Texas & Pacific Railroad,
International & Great Northern Railroad,
San Antonio & Aransas Pass Railroad,
Houston & Texas Central Railroad,
Austin & Northwestern Railroad.

Dr. Q. C. Smith, the indefatigable, as chairman of Committee on Hotels and Accommodations, informs the JOURNAL that he has arranged for reduced rates at the hotels and principal boarding houses.

